# Regulators of phagocytosis as pharmacologic targets for stroke treatment

**DOI:** 10.3389/fphar.2023.1122527

**Published:** 2023-08-02

**Authors:** Jian Cheng, Wei Wang, Yiqing Xia, Yi Li, Jia Jia, Guodong Xiao

**Affiliations:** ^1^ Clinical Research Center of Neurological Disease, The Second Affiliated Hospital of Soochow University, Suzhou, China; ^2^ Jiangsu Key Laboratory of Neuropsychiatric Diseases, Institute of Neuroscience, Soochow University, Suzhou, China; ^3^ Department of Pharmacy, The First Affiliated Hospital of Soochow University, Suzhou, China; ^4^ Academy of Pharmacy, Xi’an Jiaotong-Liverpool University, Suzhou, China; ^5^ Jiangsu Key Laboratory of Neuropsychiatric Diseases, College of Pharmaceutical Sciences, Soochow University, Suzhou, China; ^6^ Suzhou Clinical Research Center of Neurological Disease, Department of Neurology, The Second Affiliated Hospital of Soochow University, Suzhou, China

**Keywords:** phagocytosis, stroke, brain injury and repair, microglia, macrophage

## Abstract

Stroke, including ischemic and hemorrhagic stroke, causes massive cell death in the brain, which is followed by secondary inflammatory injury initiated by disease-associated molecular patterns released from dead cells. Phagocytosis, a cellular process of engulfment and digestion of dead cells, promotes the resolution of inflammation and repair following stroke. However, professional or non-professional phagocytes also phagocytose stressed but viable cells in the brain or excessively phagocytose myelin sheaths or prune synapses, consequently exacerbating brain injury and impairing repair following stroke. Phagocytosis includes the smell, eating and digestion phases. Notably, efficient phagocytosis critically depends on phagocyte capacity to take up dead cells continually due to the limited number of phagocytes vs. dead cells after injury. Moreover, phenotypic polarization of phagocytes occurring after phagocytosis is also essential to the proresolving and prorepair properties of phagocytosis. Much has been learned about the molecular signals and regulatory mechanisms governing the sense and recognition of dead cells by phagocytes during the smell and eating phase following stroke. However, some key areas remain extremely understudied, including the mechanisms involved in digestion regulation, continual phagocytosis and phagocytosis-induced phenotypic switching following stroke. Here, we summarize new discoveries related to the molecular mechanisms and multifaceted effects of phagocytosis on brain injury and repair following stroke and highlight the knowledge gaps in poststroke phagocytosis. We suggest that advancing the understanding of poststroke phagocytosis will help identify more biological targets for stroke treatment.

## 1 Introduction

Stroke, including ischemic and hemorrhagic stroke, is associated with a high risk of disability and mortality (Magid-Bernstein et al., 2022; Wang et al., 2022). Stroke results in profound death of brain cells as well as blood-derived cells, such as erythrocytes, which leak from ruptured cerebral blood vessels after hemorrhagic stroke. The persistent existence of dead cells/cell debris in the brain triggers uncontrolled neuroinflammation that causes secondary damage and tempers recovery. Phagocytosis, one type of endocytosis, is the process of uptake of large particulates, such as dead cells and bacteria, and is considered the cellular eating process (Pathak et al., 2023). Therefore, phagocytic clearance of dead cells and cellular debris prevents inflammation and contributes to tissue repair and remodeling following injury (Morioka et al., 2019; Doran et al., 2020). Thus, phagocytosis is assumed to be beneficial since it resolves inflammation via clearance of dead cells and harmful debris, such as myelin debris. However, mounting evidence suggests that excessive phagocytosis is detrimental following stroke. For instance, phagocytic clearance of stressed but still viable neurons in the peri-infarct zones exacerbates neuronal loss, thereby resulting in delayed brain atrophy following stroke (Neher et al., 2013). Thus, the existence of both beneficial and deleterious effects adds to the complexity of phagocytosis in stroke.

Brain-resident microglia and peripheral macrophages infiltrating the brain following stroke are professional phagocytes in the brain following stroke. These professional phagocytes play essential roles in engulfing harmful dead cells/cell debris, orchestrating neuroinflammation and restoring homeostasis following stroke. Moreover, increasing evidence suggests that the phagocytosis of dead cells/cell debris following cerebral injury is not limited to professional phagocytes. Astrocytes become reactive following stroke, and reactive astrocytes, particularly those in ischemic penumbra regions, also exert phagocytic functions to clear a variety of cellular debris, including debris of degenerating neurons, pre- and postsynapses and myelin following ischemic stroke (Morizawa et al., 2017; Wan et al., 2022). Interestingly, differences in spatiotemporal phagocytosis patterns likely exist between phagocytic astrocytes and microglia (Morizawa et al., 2017). Thus, professional and non-professional phagocytes may work cooperatively to clear dead cells and debris. This lends another layer of complexity to phagocytosis following stroke. Unfortunately, a comprehensive understanding of the mechanisms underlying the cooperation between microglial and astrocytic phagocytosis following stroke is lacking.

Compared to the large number of dead cells generated under pathological conditions, the number of phagocytes is rather limited (Park et al., 2011; Wang et al., 2017). As expected, it has been reported that a relatively small number of phagocytic microglia/macrophages surround large volumes of hematoma at 3 days following intracerebral hemorrhage (Yan et al., 2022a). Thus, efficient phagocytosis critically depends on the capacity of a single phagocyte to take up dead cells continually (Park et al., 2011; Wang et al., 2017; Mehrotra and Ravichandran, 2022). Phagocytosis is a multistep process, and the factors governing continual phagocytosis include each step of phagocytosis. The first step of phagocytosis is the smell phase. During this phase, dead cells release “find-me” signaling molecules that are used by phagocytes to sense and locate dead cells. The second step is the eating phase, during which phagocytes specifically recognize and bind ligands on dead cells via phagocytic receptors and ingest dead cells. The third phase is the digestion phase. During this step, phagocytes digest the corpse of dead cells and its contents, resulting in a multifold increase in the intracellular amounts of carbohydrates, nucleotides, lipids and proteins. Mounting evidence suggests that the digestion phase is critical to continual phagocytosis, since the most severe challenge for phagocytes to maintain metabolic homeostasis is how to rapidly and efficiently process the almost doubled intracellular mass following phagocytosis (Han and Ravichandran, 2011; Mehrotra and Ravichandran, 2022). Although there is great progress in the understanding of the sense and recognition of dead cells by phagocytes during the smell and eating phase following stroke, how corpse digestion and continual phagocytosis are regulated following stroke is extremely understudied. Emerging evidence suggests that phagocytosis itself induces the proresolving and prorepair phenotype switch of phagocytes. However, we currently lack an in-depth understanding of the molecular mechanisms underlying the phenotypic switch of phagocytes following stroke. In this review, we review the up-to-date literature on the key molecular mechanisms and multilayered effects of phagocytosis on brain injury and repair following stroke. In particular, we highlight these knowledge gaps in poststroke phagocytosis, which have not yet been reviewed. We suggest that advancing the understanding of these knowledge gaps will accelerate the identification of novel biological targets for stroke therapy.

## 2 Phagocytic signaling following stroke

### 2.1 Find-me signals involved in poststroke phagocytosis

**FIGURE 1:**
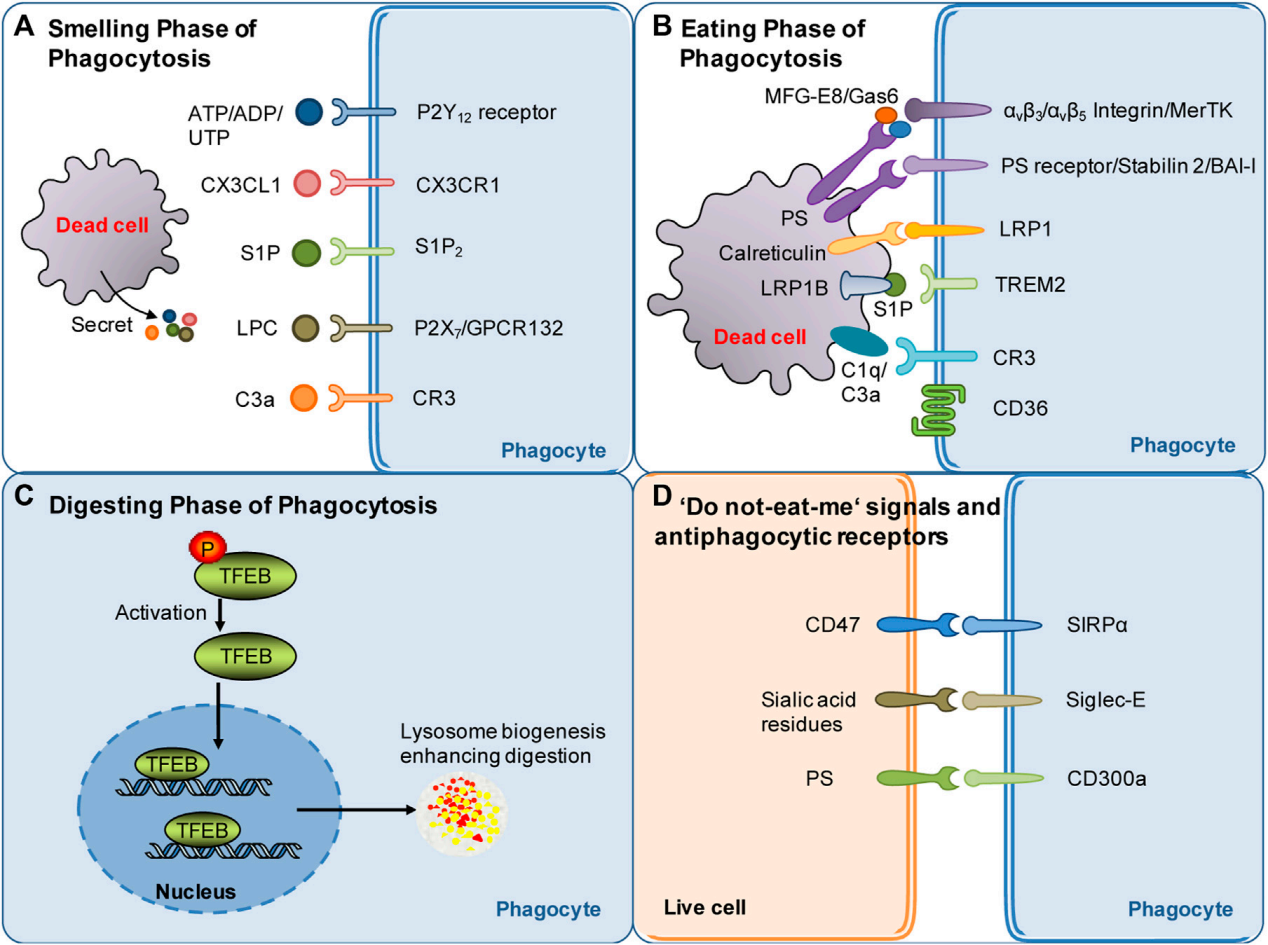
Summary of molecular targets involved in poststroke phagocytosis. **(A)** Molecular signals governing the sense of dead cells by phagocytes during the smell phase following stroke. Nucleotides, CX3CL1, S1P, LPC and other molecular signals released by dying/dead brain cells serve as find-me signals and chemoattract phagocytes following stroke. **(B)** Molecular signals governing the recognition of dead cells by phagocytes during the eating phase following stroke. Eat-me signals expressed by dead cells, such as PS flipped and exposed on the outer layer of the cell membrane, can be recognized by specific receptors of phagocytes. Opsonins, such as MFG-E8 and Gas6, associate with dead cells and strengthen the recognition of dead cells by phagocytic receptors. **(C)** Mechanisms involved in digestion modulation following stroke: current evidence suggests that TFEB activation may play an essential role in digestion regulation following stroke. **(D)** Do not-eat-me signals: one of the mechanisms through which phagocytosis is regulated. Do not-eat-me signals are recognized by antiphagocytic receptors on phagocytes, such as CD47, and inhibit phagocytosis following stroke.

The smell phase is the first step of phagocytosis. During this phase, dead or stressed cells secrete soluble mediators, termed “find-me” signals (Figure 1A), such as chemokines fractalkine CX3CL1 (Truman et al., 2008), lysophosphatidylcholine (Lauber et al., 2003; Gude et al., 2008), sphingosine-1-phosphate, and complement components (Surugiu et al., 2019) and the nucleotides ATP and UTP (Elliott et al., 2009; Chekeni et al., 2010). On the one hand, the “find-me” signaling mediators chemoattract phagocytes to migrate toward dead cells. On the other hand, these mediators induce cytoskeletal changes and promote the expression of phagocytic receptors and the digestion machinery in phagocytes, which prepares the phagocytes for subsequent engulfment and digestion (Medina and Ravichandran, 2016; Medina et al., 2020). In stroke models, dead/stressed neurons release the find-me signal lysophosphatidylcholine (LPC) by enhancing secretory phospholipase A2 group X. LPC chemoattracts microglia via G protein-coupled receptor 132 (GPCR132) or P2X purinoreceptor 7 (P2X_7_) (Inose et al., 2015) (Figure 1A). Dead/stressed neurons also secrete CX3CL1, which chemoattracts microglia via the fractalkine receptor (CX3CR1) (Harrison et al., 1998; Soriano et al., 2002). Notably, CX3CL1 knockout mice display less infarct damage following 22 h of reperfusion after transient cerebral ischemia (Soriano et al., 2002). Sphingosine-1-phosphate (S1P) likely acts as a finding-me signal via S1P receptor 2 (S1P_2_) since the blockade of S1P_2_ attenuates microglial recruitment and activation following stroke (Sapkota et al., 2019) (Figure 1A). The complement component C3a released from dying cells also chemoattracts microglia following stroke, while inhibition of the C3a receptor (CR3) with an antagonist for 2 days suppresses microglial recruitment and subsequent phagocytosis in a mouse cerebral ischemia model (Surugiu et al., 2019) (Figure 1A). In addition, knockout of CR3 reduces white matter injury by dampening microglia-mediated excessive phagocytosis of intact myelin at day 28 after ischemia in a mouse model (Zhang et al., 2020a).

Dying or stressed neurons also secrete nucleotides such as ATP and ADP, which chemoattract microglia via the P2Y_12_ receptor (Webster et al., 2013) (Figure 1A). Consistently, knockout or inhibition of the P2Y_12_ receptor suppresses microglial migration and clustering around damaged neurons, consequently decreasing damage at 3 days following transient cerebral ischemia (Webster et al., 2013). Find-me signals contribute to the anti-inflammatory properties of phagocytes (Medina and Ravichandran, 2016; Medina et al., 2020). It should be emphasized that nucleotides released in small amounts from cells during the very early stage of death (when cells are still intact) act as “find-me” signals, which are distinguishable from ATP released at high extracellular concentrations after complete cell lysis (Mehrotra and Ravichandran, 2022). Extracellular ATP at high concentrations is well known to be proinflammatory. However, recent research has suggested that pannexin-1-mediated release of ATP from viable cells is critical for suppressing airway inflammation (Medina et al., 2021). Notably, an inhibitor of pannexin-1 inhibits ATP release, suppresses neuroinflammation and improves outcome at 3–5 days postinjury in a mouse model of traumatic brain injury, suggesting that pannexin-1-mediated release of ATP contributes to neuroinflammation after brain injury (Garg et al., 2018). Thus, how pannexin-mediated ATP modulates phagocytosis and impacts neuroinflammation after stroke remains to be investigated.

### 2.2 Eat-me signals involved in poststroke phagocytosis

The exposure of “eat-me” signals on the cell surface determines whether dead cells can be recognized by phagocytes. The “eat-me” signals are recognized by a cohort of phagocytic receptors. The most potent “eat-me” signal phosphatidylserine (PS) is evolutionarily conserved and present on the outer leaflet of the plasma membrane of apoptotic cells (Fadok et al., 1992). Conversely, PS is on the inner leaflet of the plasma membrane in live cells. PS asymmetry is actively maintained via an energy-dependent mechanism by specific enzymes such as flippases and scramblases (Suzuki et al., 2013b; Suzuki et al., 2014; Segawa and Nagata, 2015; Arashiki et al., 2016). PS exposure causes phagocytosis of dead cells either directly through PS receptors or indirectly through adaptor proteins (opsonins) (Figure 1B). In dying cells, caspase 3 induces the cleavage and inactivation of flippases and simultaneously activates scramblases (Suzuki et al., 2013a). TMEM16F is a calcium-activated phospholipid scramblase. The level of TMEM16F protein is increased, and reversible PS exposure is confirmed in neurons following ischemic stroke. Inhibition of PS exposure by knocking down TMEM16F in neurons dampens phagocytosis, rescues stressed but viable neurons in the penumbra 3 days after ischemia, and consequently decreases infarct damage and improves functional recovery (Zhang et al., 2020b). Notably, PS exposure also occurs in erythrocytes leaked into the parenchyma following intracerebral hemorrhage (ICH). Clearance of erythrocytes by microglia/macrophages via PS-dependent mechanisms promotes hematoma resolution and facilitates functional recovery following ICH (Chang et al., 2018; Yan et al., 2022a). Notably, elevated calcium or oxidants or a decrease in ATP also drives reversible PS exposure on the cell surface of stressed but still viable neurons following sublethal cellular stimuli (Neher et al., 2011). This induces phagocytes to engulf stressed but viable neurons, consequently leading to delayed neuronal death, brain atrophy and behavioral deficits at 28 days after stroke (Neher et al., 2012; Neher et al., 2013). For instance, subtoxic levels of glutamate induce neurons to reversibly expose PS on their surface and subsequently induce phagocytosis of stressed but viable neurons by microglia. Consequently, this kind of phagocytosis can be attenuated by blocking PS exposure (Neher et al., 2013).

Although PS is the major “eat-me” signal, a cohort of other “eat-me” signals also contribute to the recognition of dead cells by phagocytes (Mehrotra and Ravichandran, 2022). Other “eat-me” signals include calreticulin (Lauber et al., 2004; Arandjelovic and Ravichandran, 2015). Calreticulin, as an alternative “eat-me” signal, engages low-density lipoprotein receptor-related protein 1 (LRP1) on the cell surface of microglia/macrophages to initiate phagocytosis (Gardai et al., 2005) (Figure 1B). Calreticulin normally resides in the endoplasmic reticulum in healthy cells. It translocates to the cell surface upon endoplasmic reticulum stress, apoptosis, or proinflammatory signaling (Cockram et al., 2021). The calreticulin/LRP phagocytic signaling pathway mediates microglial phagocytosis of lipopolysaccharide- or Aβ-stressed neurons (Fricker et al., 2012). More relevantly, the signaling pathway likely plays a role in heme scavenging following ICH (Wang et al., 2018). The role of calreticulin in poststroke phagocytosis deserves further investigation. Notably, not all dead cells expose the same set of “eat-me” signals. Cell-exposed PS and a subset of other “eat-me” signals can trigger recognition by specific receptors on phagocytes (Mehrotra and Ravichandran, 2022).

### 2.3 Phagocytic receptors and opsonins involved in poststroke phagocytosis

PS exposed on the cell surface can be recognized by the receptors of phagocytes that bind PS directly, such as phosphatidylserine receptor (PSR), the T-cell immunoglobulin and mucin (TIM) family members, Stabilin2, and brain angiogenesis inhibitor I (BAI-I) (Fadok et al., 2000; Kobayashi et al., 2007; Miyanishi et al., 2007; Park et al., 2007; Park et al., 2008) (Figure 1B). PS can also be recognized via adaptor proteins such as milk fat globule-epidermal growth factor 8 (MFG-E8) and growth arrest-specific gene 6 (Gas6), which link to integrin or tyrosine protein kinase Mer (MerTK) receptors, respectively, on the phagocyte surface (Hanayama et al., 2002; Leonardi-Essmann et al., 2005; Brown and Neher, 2014) (Figure 1B). Notably, different cells express different sets of phagocytic receptors, which may depend on the stages of phagocytosis and tissue context. The binding of dead cells to phagocytic receptors triggers a complex array of cytoskeletal rearrangements, facilitating cargo engulfment. The signaling mechanism, the proteins involved and the kinetics of the process are highly variable following stroke and deserve further investigation.

Macrophages/microglia can also recognize and bind to apoptotic cells via scavenger receptors (Figure 1B). CD36 is a class B scavenger receptor. CD36 expression is low in the normal brain and significantly enhanced following stroke, mainly in macrophages in the brain (Kim et al., 2012). CD36 displays high affinity for many ligands, including apoptotic cells, and is well known to mediate the phagocytosis of erythrocytes following ICH (Zhao et al., 2009). CD36 knockout delays hematoma absorption and enlarges hematoma volumes at 5 days following ICH (Fang et al., 2014). Upregulating CD36 expression in microglia/macrophages via activation of the transcription factor nuclear factor-erythroid 2 p45-related factor 2 (Nrf2) promotes erythrocyte clearance at 7 or 10 days following ICH (Zhao et al., 2015). Bexarotene, a selective retinoid X receptor (RXR) agonist, also promotes erythrocyte phagocytosis, possibly via a mechanism related to CD36 upregulation 7 days following ICH (Chang et al., 2020). In contrast to the beneficial role of CD36 in ICH models, genetic deletion studies have shown that CD36 aggravates acute brain injury at 3 days after cerebral ischemia (Cho et al., 2005). However, CD36 may play a differential role in the recovery phase following cerebral ischemia. A study suggested that CD36 in macrophages mediates phagocytosis during the recovery phase and likely plays a reparative role via the resolution of inflammation following ischemic stroke (Woo et al., 2016). In conclusion, the scavenger receptor CD36, as an essential phagocytic receptor, exerts complex effects following stroke.

Opsonins associate with dead cells and thereby make dead cells visible to specialized phagocyte receptors. MFG-E8 and Gas6 are opsonins that are normally extracellular proteins. MFG-E8 recognizes and binds with PS exposed on neurons and therefore drives the microglial phagocytosis of PS-exposed neurons via the vitronectin receptors α_v_β_3_ or α_v_β_5_ on phagocytes (Neniskyte and Brown, 2013) (Figure 1B). Alternatively, PS exposed on neurons can be recognized by Gas6, which then induces microglial phagocytosis of neurons via the phagocytic receptor MerTK (Fourgeaud et al., 2016) (Figure 1B). The expression of MFG-E8 and MerTK is transiently enhanced in microglia/macrophages at 3–7 days after stroke, and knockout of either protein prevents delayed neuronal loss and brain atrophy and improves long-term functional deficits at 28 days after focal cerebral ischemia (Neher et al., 2013). This suggests that neuronal PS exposure drives phagocytosis of stressed neurons via opsonins after stroke and that blocking signaling is beneficial following cerebral ischemia.

The role of complement proteins such as opsonins in poststroke phagocytosis has been demonstrated by a recent study, which showed that targeted complement inhibition in the ischemic area salvages stressed neurons and inhibits neuroinflammation after ischemic stroke (Alawieh et al., 2018). Complement components, including C1q and C3, can be produced by microglia. The complement component C1q, as an opsonin, binds to eat-me signals, such as surface PS and calreticuline, to strengthen phagocytic recognition and promote phagocytosis (Cockram et al., 2021). C1q may also interact with the phagocytic receptor complement receptor 3 on the phagocyte surface to induce phagocytosis (Linnartz et al., 2012) (Figure 1B). C1q, as an opsonin, has an important role in synapse pruning. C1q binds to neurites after enzymatic removal of sialic acid residues from the neuronal glycocalyx, and the elimination of desialylated neurites is mediated by the complement receptor CR3 (Linnartz et al., 2012). Notably, reducing C1q levels by inhibiting the classical complement pathway attenuates brain damage at 2 days after hypoxia in a mouse model of hypoxic ischemic encephalopathy (Kumar et al., 2021). Moreover, the serum levels of C1q correlate with the severity of neurological deficits and infarct sizes in human patients with ischemic stroke (Wang D D et al., 2020). These results suggest that opsonin C1q has great impacts on stroke outcomes.

The conversion of C3 to C3a and C3b is essential for complement-opsonin activation (Kumar et al., 2021). C3a recruits and activates microglia, whereas C3b promotes microglial phagocytosis via the CR3 receptor (Yu et al., 2022) (Figure 1B). Complement C3 inhibition specifically at synapses inhibits synaptic engulfment by microglia and reduces synapse loss in a mouse model of multiple sclerosis (Werneburg et al., 2020). More relevantly, C3a peptide treatment reduced tissue loss at 22 days after hypoxia–ischemia and improved memory impairment at 41 days after neonatal hypoxia–ischemia in wild-type mice but not in CR3-deficient mice (Jarlestedt et al., 2013), suggesting that complement C3a (*C3a*) acts through the receptor of *C3a* to protect against ischemic injury.

A recent study reported that S1P released from microglia may also function as a novel opsonin that engages triggering receptor expressed on myeloid cells 2 (TREM2) on microglia to promote microglial phagocytosis (Figure 1B). The study showed that microglia released apolipoprotein E-loaded S1P, which bound to apoptotic neurons via low-density lipoprotein receptor related protein 1B (LRP1B). Then, S1P binds to TREM2 on microglia to promote microglial phagocytosis of apoptotic neurons and thereby protect against ischemic brain injury at 48 h after cerebral ischemia (Xue et al., 2022). These results suggest complex roles of S1P in poststroke phagocytosis and pathology.

### 2.4 Do not eat me’ signals and antiphagocytic receptors

The exposure of “do not eat-me” signals on viable cells suppresses phagocytosis (Segawa et al., 2011) Numerous “do not eat-me” molecules have been discovered. These “do not eat-me” signaling molecules bind “anti-phagocytic receptors” on phagocytes to inhibit phagocytosis of live cells by phagocytes. For instance, CD47 on live cells binds to signal-regulatory protein-α (SIRPα) on macrophages (Figure 1D). SIRPα/CD47 binding induces tyrosine phosphorylation of the cytoplasmic domain of SIRPα, leading to the recruitment and activation of the phosphatases SHP1/2. SHP1/2 activation inhibits phagocytosis by suppressing non-muscle myosin IIA (Tsai and Discher, 2008). Knockout of the CD47 gene confers protection at 24 and 72 h after ischemia in a murine model of transient focal cerebral ischemia (Jin et al., 2009). Consistently, SIRPα deletion also confers robust neuroprotection at 24 h following cerebral ischemia (Wang et al., 2012). Thus, current evidence suggests that inhibiting SIRPα/CD47 signaling is neuroprotective following focal cerebral ischemia.

CD47 also acts as a “do not eat me signal” on erythrocytes and normally inhibits erythrophagocytosis via SIRPα expressed on phagocytes (Ni et al., 2016). In nude mice injected with blood from CD47 knockout or wild-type mice, CD47 knockout blood had quicker hematoma absorption than wild-type blood at 3 days after ICH (Ni et al., 2016). A CD47 blocking antibody also accelerated hematoma clearance in both young and aged mice (Jing et al., 2019; Tao et al., 2020). CD47 levels within the hematoma decreased with time in a pig ICH model (Cao et al., 2016). CD47 is also involved in erythrocyte clearance in other models of intracranial hemorrhage. In a rat model of intraventricular hemorrhage (IVH), the CD47 blocking antibody accelerated absorption of the intraventricular clot and reduced early erythrolysis at day 3 after IVH (Ye et al., 2021). Collectively, emerging evidence suggests that the “do not eat me” signal of CD47 delays erythrophagocytosis following hemorrhagic stroke.

Healthy cells have sialic acid residues that are integrated into glycoproteins and glycolipids residing on the cell surface (Figure 1D). Sialylation of glycoproteins and glycolipids inhibits phagocytosis via sialic acid-binding immunoglobulin-like lectins (Siglec) on the phagocyte cell surface, whereas desialylated glycoproteins residing on the cell surface promote phagocytosis by amplifying “eat-me” signals (Yu et al., 2022). Siglec-E is expressed in the brain, particularly on the surface of microglia. Genetic deletion of Siglec-E exacerbated neuronal death induced by oxygen-glucose deprivation in mouse primary cortical cultures, a mixed culture containing both neurons and glial cells. Moreover, compared to wild-type animals, Siglec-E knockout mice display more severe neurological deficits and larger infarct sizes at day 3 after cerebral ischemia (Li et al., 2022). Therefore, further investigation is needed to explore whether Siglec-E contributes to stroke outcomes by serving as a “do not eat me signal.”

Recently, the phosphatidylserine receptor CD300a was identified to function as an anti-phaocytic receptor during poststroke phagocytosis (Figure 1D). CD300a is highly expressed on infiltrated myeloid cells, including monocytes. CD300a can act through the CD300b-DNAX-activation protein 12 signaling pathway to inhibit phagocytosis of apoptotic cells. Deletion of CD300a enhanced phagocytosis by myeloid cells infiltrating the brain and consequently reduced the release of damage-associated molecular patterns from dead cells, attenuating inflammation in the penumbral region. An anti-CD300a neutralizing antibody also reduced infarction at 24 h after ischemia and ameliorated neurological deficits during the acute phase of ischemic stroke (Nakahashi-Oda et al., 2021). Therefore, current evidence suggests that “do not eat me” signals play a complex role following stroke.

## 3 Mechanisms involved in digestion regulation following stroke

The number of apoptotic cells far exceeds that of phagocytes under either physiological or pathological conditions. Thus, each phagocyte must continually ingest multiple apoptotic corpses, some of which can be very large in size. Mounting evidence suggests that the digestion of phagocytic corpse cargos is critical to continual phagocytosis, since the most severe challenge for phagocytes following phagocytosis is how to rapidly and efficiently process the almost doubled intracellular mass to maintain metabolic homeostasis (Han and Ravichandran, 2011; Mehrotra and Ravichandran, 2022). The corpse cargo, which bears membranes, cholesterol, proteins, and nucleic acids, is a metabolic burden for phagocytes. Therefore, the corpse cargo needs to be digested and subsequently metabolized via metabolic flux cycles in phagocytes. However, how digestion is regulated following stroke and how the digestion phase affects stroke outcomes are extremely understudied thus far.

Lysosomes, acidic organelles filled with numerous hydrolases, serve as recycling centers in mammalian cells. Lysosomes are responsible for breaking down endocytosis substrates, such as membranes, proteins, and lipids, into their basic components. The processing and degradation of corpse cargos involves the maturation of the early phagosome to late phagosomes with eventual fusion with the lysosomes (Boada-Romero et al., 2020; Mehrotra and Ravichandran, 2022). Transcription factor EB (TFEB) belongs to the Microphthalmia/TFE family of leucine zipper transcription factors (Irazoqui, 2020). TFEB, generally located in the cytoplasm, translocates to the nucleus if activated, where it binds to a consensus DNA sequence in the promoters of lysosomal genes, termed the coordinated lysosomal expression and regulation motif, to upregulate genes for lysosomal biogenesis and functions. Thus, TFEB coordinates the expression and regulation of lysosomes and is a master regulator of the lysosomal system (Irazoqui, 2020) (Figure 1C). Theoretically, TFEB should play an important role in the digestion phase of poststroke phagocytosis. Indeed, some publications have reported that TFEB activators and GSK-3β inhibition facilitated TFEB nuclear translocation and thus conferred neuroprotection at 24 h after cerebral ischemia/reperfusion injury (Wu et al., 2021; Zhang et al., 2022). However, it is currently not clear whether TFEB activation plays important roles in poststroke phagocytosis and thereby contributes to stroke pathology and outcomes by modulating the digestion of phagocytic cargos. This issue warrants further investigation.

Notably, the digestion of phagocytic cargos also generates harmful contents, such as free cholesterol. Thus, shifting the phenotype of phagocytes toward an anti-inflammatory, prorepair phenotype during the digestion and degradation of phagocytic cargos is another challenge for phagocytes and is essential for tissue repair and remodeling (Zhang et al., 2019; Jia et al., 2022). A recent study showed that engulfment of apoptotic erythrocytes with exposed phosphatidylserine is required for the phenotypic shifting of both murine and human phagocytic macrophages toward the reparative phenotype after ICH (Chang et al., 2018). Further investigation is needed to examine how ingestion and subsequent digestion of apoptotic erythrocytes induce the phenotypic shifting of phagocytes following stroke. In particular, it may be important to explore whether metabolic reprogramming essentially contributes to phagocytosis-mediated phenotypic polarization of phagocytes, as metabolic reprogramming has been shown to be essential for the phenotypic polarization of macrophages undergoing efferocytosis following myocardial infarction (Zhang et al., 2019). Understanding these mechanisms is essential for manipulating phagocyte phenotypes to reduce brain injury and promote recovery following stroke (Figure 1C).

## 4 Phagocytosis by non-professional phagocytic astrocytes after stroke

Initially, phagocytosis of dead cells or cell debris in the brain was thought to be limited to professional phagocytes, such as brain-resident microglia (Koizumi et al., 2007; Fourgeaud et al., 2016). Although astrocytic phagocytosis has received limited attention and the physiological consequences and mechanisms are poorly understood, increasing evidence suggests that astrocytes also exert phagocytic functions and participate in phagocytosis in the brain under both physiological and pathological conditions. For instance, astrocytes located in the optic nerve head constitutively engulf axonal materials under normal physiological conditions (Nguyen et al., 2011; Davis et al., 2014). Moreover, it has been reported that immature astrocytes actively eliminate synapses via MerTK pathways in the developing retino-geniculate system (Chung et al., 2013). Mechanistically, it has been shown that genes involved in engulfment, such as phagocytic receptors and opsonins, are enriched in astrocytes in the developing forebrain (Cahoy et al., 2008). Moreover, degenerated axons and apoptotic neurons have been detected in astrocytes in injured brains (Al-Ali et al., 1988; Loov et al., 2012). Astrocytes are highly responsive and change their phenotype into “reactive astrocytes” in response to brain damage (Anderson et al., 2016). It has been reported that astrocytes within the ischemic penumbra region are transformed into phagocytes in the adult brain following transient ischemic injury. Several molecules, such as ATP-binding cassette transporter A1 (ABCA1), multiple EGF-like domains 10 and the engulfment adaptor phosphotyrosine binding domain containing 1, have been shown to be essential for astrocytic phagocytosis. It has been reported that upregulation of ABCA1 alone is sufficient for enhancing astrocytic phagocytosis (Morizawa et al., 2017). Moreover, astrocyte-mediated phagocytosis displays a distinct spatiotemporal pattern from that mediated by microglia, i.e., astrocytic phagocytosis displays a late onset within the ischemic penumbra, while the onset of microglial phagocytosis is early within the ischemic core (Morizawa et al., 2017). Together, these findings suggest that astrocytes can perform phagocytic functions in the ischemic brain and contribute to phagocytosis and remodeling of the ischemic brain.

Brain injuries, including cerebral ischemia, generate myelin debris that contributes to neuroinflammation and oxidative stress (Arandjelovic and Ravichandran, 2015). Rapid clearance of myelin debris by phagocytes prevents detrimental effects of myelin debris, thereby facilitating brain regeneration and remodeling following brain injury (Konishi et al., 2020). However, excessive phagocytosis of myelin by astrocytes is also responsible for demyelination injury following stroke. Astrocytes are the major source of lipocalin-2 (LCN2), which is a characteristic marker for reactive astrocytes (Zamanian et al., 2012; Suk, 2016). In a mouse model of distal middle cerebral artery occlusion, LCN2 was upregulated and enriched in reactive astrocytes in non-ischemic areas of the corpus callosum. LCN2-expressing astrocytes displayed a phagocytic phenotype and took up myelin. Mechanistically, LCN2 binds to LRP1 to promote astrocytic phagocytosis. LCN2-induced myelin engulfment by astrocytes and demyelination were inhibited by LRP1 knockdown. Thus, astrocyte-mediated myelin phagocytosis may be responsible for demyelination injury in non-ischemic regions after ischemic stroke (Wan et al., 2022).

Astrocytic phagocytosis and microglial phagocytosis may play distinct roles but cooperate to perform phagocytic functions following ischemic and hemorrhagic stroke. For instance, it has been suggested that phagocytic astrocytes are likely involved in remodeling the brain microenvironment within the penumbra region, while microglia with phagocytic functions are mainly present in the ischemic core region during the early phase after stroke (Morizawa et al., 2017). By specific deletion of multiple EGF like domains 10 (MEGF10) and MerTK phagocytic receptors from microglia/macrophages or astrocytes to block the phagocytosis mediated by these cells, a recent study showed that suppressing phagocytosis mediated by microglia/macrophages or that by astrocytes reduced brain damage and improved neurobehavioral deficits at 14 days after ischemic stroke. Interestingly, inhibiting phagocytosis mediated by microglia/macrophages but not that mediated by astrocytes improved neurobehavioral outcomes at 14 days after hemorrhagic stroke in mice (Shi et al., 2021). Single-cell RNA sequencing further revealed that the genes involved in phagocytosis pathways were downregulated in astrocytes in the hemorrhagic brain vs. astrocytes in the ischemic brain. To conclude, current evidence suggests that reactive microglia and astrocytes display distinct effects in pathologically distinct stroke models and thus exert distinct actions on stroke outcomes (Shi et al., 2021).

## 5 Regulatory mechanisms of phagocytosis following stroke

**FIGURE 2:**
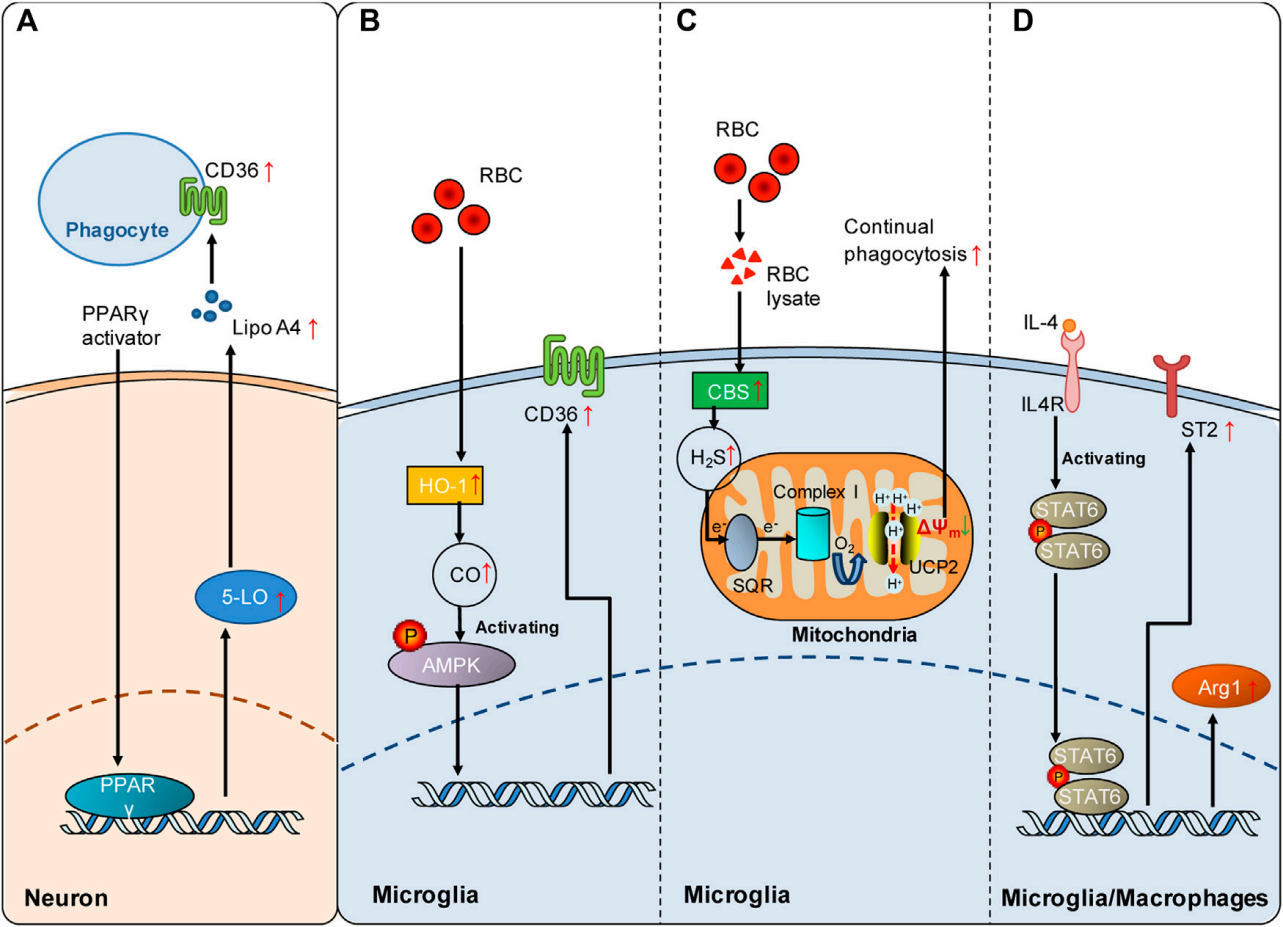
Signaling pathways involved in the regulation of poststroke phagocytosis. **(A)** PPARγ activation enhances the expression of CD36 on the cell surface of phagocytes by upregulating the 5-LO/Lipo A4 signaling pathway in neurons following ICH. **(B)** Red blood cells (RBCs) enhance HO-1-derived endogenous CO in microglia, which increases microglial expression of CD36 via AMPK activation and consequently promotes microglial phagocytosis of RBCs and hematoma clearance following SAH. **(C)** RBC lysate increases CBS-derived endogenous H_2_S in microglia, which promotes continual phagocytosis of RBCs by microglia and consequently contributes to spontaneous hematoma clearance following ICH. Mechanistically, SQR oxidation of endogenous H_2_S drives reverse electron transport at mitochondrial complex I, which generates superoxide to activate UCP2 and consequently dissipates the mitochondrial membrane potential (ΔΨ_m_). The decrease in ΔΨ_m_ is an established mechanism underlying continual phagocytosis. **(D)** The IL-4/STAT6 axis enhances erythrocyte engulfment and accelerates hematoma clearance by upregulating ST2 after ICH or confers neuroprotection by facilitating phagocytosis of dead cells via upregulated Arg1 following cerebral ischemia. Red arrows indicate the increased expression of the mediators. The green arrow indicates the decrease in mitochondrial membrane potential.

Phagocytosis includes the smell phase, eating phage and digestion phase. Mechanistically, regulation of each phase of phagocytosis has profound effects on phagocytosis. Successful regulation of phagocytosis via different modalities may represent a promising therapeutic strategy for treating stroke. However, how phagocytosis is regulated following stroke remains poorly understood. Most currently published studies focus on how to promote phagocytosis by modulating phagocytic signals during the smell and eating phases. Little attention has been given to digestion regulation. Here, we examine current evidence about the regulatory mechanisms of phagocytosis following stroke, as summarized in Figure 2.

### 5.1 Regulation of poststroke phagocytosis by PPARγ and Nrf2

Peroxisome proliferator-activated receptor-γ (PPARγ) and nuclear factor erythroid 2-related factor (Nrf 2) are the first two transcription factors identified to be involved in the regulation of phagocytic receptor expression following stroke. PPARγ displays multiple functions under pathophysiological conditions (Heming et al., 2018). PPARγ activation is an important mechanism underlying monocyte differentiation into macrophages with efficient phagocytic capacities (Cai et al., 2018). Notably, it has been shown that PPARγ activation promotes phagocytosis via the mechanism of CD36 upregulation, which is beneficial in both hemorrhagic and ischemic stroke models. PPARγ activation enhances the expression of the scavenging receptor CD36 in microglia/macrophages, facilitates the phagocytic clearance of erythrocytes, and consequently improves functional recovery in rodent models of ICH (Zhao et al., 2007). In animal models of cerebral ischemia, PPARγ activation by rosiglitazone also increases CD36 expression in microglia, which contributes to the resolution of inflammation and clearance of infiltrated neutrophils at 48 h following cerebral ischemia (Ballesteros et al., 2014). PPARγ activation likely increases CD36 expression via a 5-lipoxygenase (5-LO)-mediated mechanism. PPARγ activation induced 5-LO expression in neurons, and lipoxin A_4_, one of the products of 5-LO, and lipoxin A_4_ (Lipo A_4_) enhanced CD36 independently of PPARγ (Ballesteros et al., 2014) (Figure 2A).

The transcription factor nuclear factor erythroid 2-related factor (Nrf2) plays a crucial role in antioxidant and anti-inflammatory responses. The Nrf2 agonist sulforaphane enhances phagocytosis of erythrocytes by upregulating the expression of CD36 in microglia after ICH (Zhao et al., 2015). Consequently, promoting hematoma clearance by Nrf2 activation improved outcomes following ICH. Moreover, CDDO-EA, a novel Nrf2 activator, also protects against ischemic injury by enhancing inducible heme oxygenase (HO-1) expression and microglial polar polarization toward the M2 phenotype at 48 h after ischemic stroke in mice (Lei et al., 2021). Thus, Nrf2 activation is an important regulatory mechanism underlying CD36-mediated phagocytosis following stroke.

### 5.2 Regulation of poststroke phagocytosis by endogenous gasotransmitters

Carbon monoxide (CO) and hydrogen sulfide (H_2_S) are endogenous gasotransmitters displaying essential roles in multiple pathophysiological processes, along with the first identified gasotransmitter nitric oxide. Interestingly, both CO and H_2_S have been shown to be involved in regulating phagocytosis following hemorrhagic stroke. Spontaneous hematoma resolution occurs both in human patients and animal models following cerebral hemorrhage. Theoretically, erythrophagocytosis plays an important role in spontaneous hematoma resolution. Nevertheless, the mechanisms underlying spontaneous hematoma resolution are poorly understood.

Subarachnoid hemorrhage (SAH) is a severe form of stroke that carries a mortality rate of 50%. Following SAH, heme released from extraversated erythrocytes functions as a disease-associated molecular pattern. Free heme is metabolized by heme oxygenase (HO), leading to the production of CO. In a mouse model of SAH, the expression of inducible HO (HO-1) in microglia is enhanced following SAH, which consequently attenuates neuronal cell death and vasospasm, improves cognitive function and promotes clearance of cerebral blood burden (Schallner et al., 2015). Inhalation of CO after SAH reduced injury by enhancing erythrophagocytosis in mice with HO-1 deletion in microglia. Clinical data from human SAH patients further revealed that HO-1 activity was markedly increased in cerebrospinal fluid (CSF). Moreover, cisternal hematoma volume was negatively correlated with HO-1 activity in the CSF of these patients. In conclusion, microglial generation of CO by HO-1 is a regulatory mechanism underlying erythrophagocytosis following SAH (Schallner et al., 2015). Mechanistically, CO enhanced microglial phagocytosis of erythrocytes by activating AMP-activated protein kinase (AMPK) to upregulate the expression of the phagocytic receptor CD36 in microglia following SAH (Kaiser et al., 2020) (Figure 2B). Collectively, current evidence suggests that the generation of endogenous gasotransmitter CO is an important regulatory mechanism underlying erythrophagocytosis and spontaneous hematoma resolution following SAH.

H_2_S plays important roles in pathogenesis of the nervous system (Hou et al., 2017; Zhao et al., 2017). Notably, exogenous and endogenous H_2_S has been shown to protect against injuries from both ischemic and hemorrhagic stroke (Wang et al., 2014; Zhang et al., 2017; Jia et al., 2020; Yan et al., 2022b). Recently, it was reported that ICH enhanced the expression of the H_2_S synthase cystathionine β-synthase (CBS) and CBS-derived H_2_S in brain-resident phagocytic microglia (Yan et al., 2022a). Notably, endogenous H_2_S derived from CBS in microglia promoted continual phagocytosis of erythrocytes by microglia *in vitro* and contributed to spontaneous hematoma resolution at 5 days and 14 days following ICH in a mouse model. Mechanistically, oxidation of CBS-derived endogenous H_2_S by sulfide-quinone oxidoreductase (SQR) initiated reverse electron transfer at mitochondrial complex I, resulting in enhanced superoxide production. Complex I-derived superoxide subsequently activated uncoupling protein 2 (UCP2) to promote erythrocyte phagocytosis by microglia (Figure 2C). UCP2 has been reported to be an essential mediator of continual phagocytosis by reducing mitochondrial membrane potential (Park et al., 2011). Moreover, hyperhomocysteinemia, an established stroke risk factor, is found to impair ICH-enhanced CBS expression and delay spontaneous hematoma clearance, while administration of an H_2_S donor facilitated hematoma resolution in mice with hyperhomocysteinemia. The study showed that enhancing the production of endogenous H_2_S following ICH is an endogenous regulatory mechanism underlying erythrophagocytosis and spontaneous hematoma resolution following ICH. For the first time, the study suggested a mechanism that regulates continual phagocytosis following stroke.

### 5.3 Regulation of poststroke phagocytosis by STAT6

As a member of the signal transducer and activator of transcription family, signal transducer and activator transcription 6 (STAT6) is principally activated by interleukin-13 (IL-13) and interleukin-4 (IL-4) (Quelle et al., 1995). STAT6 exerts multiple functions in lymphocytes and myeloid cells (Goenka and Kaplan, 2011). The expression of STAT6 is enhanced, and its related signaling cascades are activated following both ischemic and hemorrhagic strokes, which mechanistically promote microglial/macrophage phagocytosis and functional outcomes. In animal models of cerebral ischemia, the STAT6/arginase 1 (STAT6/Arg1) pathway contributes to phagocytosis of dead/dying cells by microglia and macrophages, and upregulating STAT6/Arg1 signaling reduces brain infarction and facilitates long-term functional recovery at 3 and 7 days following ischemic stroke in mice (Cai et al., 2019). STAT6 is also an essential mediator of erythrocyte phagocytosis after ICH, and the IL-4/STAT6 pathway enhances long-term recovery in models of ICH (Xu et al., 2020). Enhancing the expression of interleukin-1 receptor-like 1 (ST2) is likely the key downstream mechanism underlying hematoma clearance enhanced by IL-4/STAT6 following ICH (Figure 2D).

## 6 The role of phagocytosis in brain injury and repair after stroke

Phagocytosis plays dynamic and multifaceted roles in brain injury and repair following stroke. One important consequence of poststroke phagocytosis of dead/dying cells and cell debris is the inhibition of neuroinflammation by preventing potentially cytotoxic effects of the intracellularly released cellular contents (Fukumoto et al., 2019). Microglia, brain-resident phagocytes, are reported to be responsible for more phagocytosis of extraverted erythrocytes after ICH and SAH than infiltrating macrophages (Schallner et al., 2015; Yan et al., 2022a). Phagocytosis of extraverted erythrocytes limited neurotoxic effects derived from extraverted red blood cells and accelerated hematoma resolution and functional recovery after hemorrhagic stroke (Zhao et al., 2009; Yan et al., 2022a). Thus, enhancing microglial phagocytosis is beneficial following hemorrhagic stroke. In addition, it has also been reported that the phagocytic activity of resident microglia is predominant over that of infiltrating macrophages following transient focal cerebral ischemia (Schilling et al., 2005). Proinflammatory brain injury at the acute phase of stroke is partly attributed to the infiltration and accumulation of neutrophils in the ischemic brain (Jickling et al., 2015). Microglia can phagocytose infiltrating neutrophils and monocytes, thereby attenuating neutrophil-mediated brain injury following stroke (Neumann et al., 2008). For instance, eliminating microglia or impairing microglial function leads to the accumulation of neutrophils in the perivascular spaces and parenchyma, accordingly enlarging the ischemic lesion (Otxoa-de-Amezaga et al., 2019). Thus, enhancing microglial phagocytosis of infiltrating neutrophils is protective following ischemic stroke. Following stroke, infiltrating myeloid cells, such as macrophages, are transformed into the phenotype with enhanced phagocytosis, as indicated by the enrichment of genes involved in phagocytosis in these cells (Chang et al., 2018; Wang R et al., 2020). CD300a is an antiphagocytic receptor highly expressed on infiltrated myeloid cells. As we discussed above, CD300a deletion or CD300a neutralizing antibody attenuates inflammation and ameliorates neurological deficits during the superacute phase of ischemic stroke (Nakahashi-Oda et al., 2021). Thus, promoting infiltrating myeloid cell-mediated phagocytosis may also exert beneficial effects following stroke.

In contrast, phagocytosis of stressed yet viable neurons may exacerbate injury following stroke. After cerebral ischemia, neuronal PS exposure on the outer membrane is triggered by the calcium-activated phosphatidylserine scramblase TMEM16F (Zhang et al., 2020b). PS exposure initiates the phagocytosis of stressed but salvageable neurons by microglia, and the process may induce excessive neuronal loss that leads to brain atrophy in the period of 4–24 h after cerebral ischemia (Brown and Neher, 2014; Zhang et al., 2020b). Moreover, microglia may promote vessel disintegration and impair blood‒brain barrier integrity by upregulating CD68 expression to enhance phagocytosis of endothelial cells. This process, in turn, further facilitates the infiltration of peripheral immune cells into the brain parenchyma and exacerbates brain injury 24 h post cerebral ischemia (Jolivel et al., 2015).

The effects of phagocytosis on long-term repair and functional recovery after stroke are extremely understudied. The effects of phagocytosis of viable cells, synapses and myelin sheaths on long-term stroke outcomes remain unclear. Whether the clearance of stressed but viable cells facilitates long-term rewiring deserves further investigation. Synapses in ischemic regions display enhanced turnover rates upon contact with microglia, suggesting that microglia are engaged to prune synapses after stroke (Wake et al., 2009). Reactive microglia and astroglia have been reported to hinder brain repair by engulfing synapses, and inhibition of this type of synapse phagocytosis displays beneficial effects, including recusing synapse loss and improving neurobehavioral deficits (Shi et al., 2021). Notably, inhibiting phagocytosis mediated by microglia/macrophages or phagocytosis by astrocytes attenuated brain damage and improved neurobehavioral outcomes after ischemic stroke. In contrast, inhibiting phagocytosis by microglia/macrophages but not by astrocytes improved neurobehavioral outcomes following hemorrhagic stroke (Shi et al., 2021). This suggests that reactive microglia and astrocytes may play distinct roles in engulfing synapses and thereby exert distinct actions on long-term outcomes after hemorrhagic and ischemic stroke (Shi et al., 2021).

Excessive phagocytosis of myelin sheaths by astrocytes may also accelerate demyelination with detrimental consequences following stroke (Wan et al., 2022). In addition, microglia not only play an important role in sculpting myelination via myelin phagocytosis in developing brains (Hughes and Appel, 2020) but are also responsible for myelin damage due to their excessive engulfment of myelin sheaths after stroke (Zhang et al., 2020a). Notably, after phagocytosing myelin debris enriched in cholesterol, microglia can contribute to oligodendrocyte-mediated remyelination and white matter repair by synthesizing sterols in a disease model of multiple sclerosis (Berghoff et al., 2021). Whether microglia can contribute to poststroke repair and thereby facilitate long-term functional recovery through the same mechanism deserves further investigation.

In conclusion, the effects of poststroke phagocytosis on stroke outcomes are context-dependent and cell type dependent. It represents a challenge to balance phagocytosis effects to establish a milieu favorable for brain repair and functional recovery. Notably, a recent study suggests that normal or excessive phagocytic functions may be related to the phenotypic polarization of phagocytes: anti-inflammatory microglia/macrophages perform normal phagocytic function, while proinflammatory microglia/macrophages display excessive phagocytic function (Wang K et al., 2022). In particular, a study showed that deletion of salt-induced kinase 3 promoted functional recovery at 35 days following cerebral ischemia in mice by enhancing normal phagocytosis of myelin debris but attenuating excessive phagocytosis of the non-damaged myelin sheath, which was accompanied by reduced expression of proinflammatory markers (Wang K et al., 2022). Further research is needed to confirm whether phagocytosis functions are determined by specific phagocyte phenotypes and to explore how to manipulate phagocyte phenotypes to promote protective and prorepair phagocytosis.

## 7 Knowledge gap and perspectives

**FIGURE 3:**
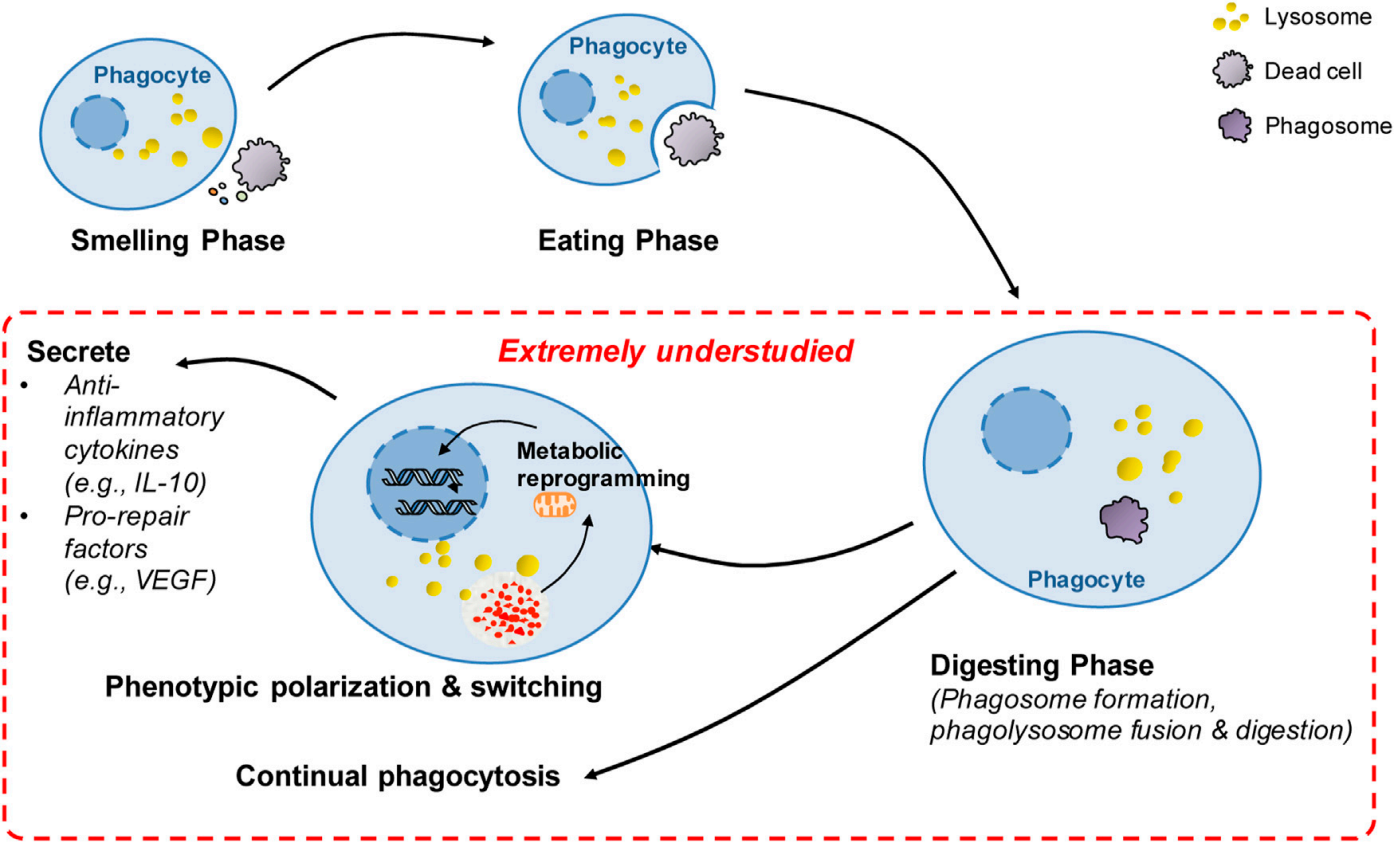
Summary of extremely understudied key areas and future directions in poststroke phagocytosis. There has been great progress in the understanding of molecular targets and regulatory mechanisms governing the sense and recognition of dead cells by phagocytes during the smell and eating phase following stroke. However, after the initial engulfment of dead cells, the key processes in poststroke phagocytosis remain extremely understudied (highlighted in red), including the mechanisms involved in digestion regulation, continual phagocytosis and metabolic reprogramming-mediated phenotypic switching following phagocytosis. These extremely understudied areas represent future directions in the stroke field.

Much has been learned about the molecular mechanisms governing the sense and recognition of dead cells by phagocytes during the smell and eating phase following stroke. However, there are still knowledge gaps in our understanding of poststroke phagocytosis (Figure 3), as we discussed below.

### 7.1 How is the digestion phase regulated following stroke?

Phagocytosis includes the smell, eating and digestion phases. Most studies on poststroke phagocytosis focus on the smell and eating phase, especially on the “find-me” and “eat-me” signals and the corresponding receptors that enable the sense and recognition of dead/dying cells by phagocytes. How the digestion phase is regulated and how digestion impacts phagocytosis and stroke outcomes are extremely understudied. Indeed, the most severe challenge for phagocytes following engulfment of dead cells is how to rapidly and efficiently process the almost doubled intracellular mass to maintain metabolic homeostasis (Han and Ravichandran, 2011; Mehrotra and Ravichandran, 2022). Mounting evidence suggests that the digestion phase is critical to phagocytosis. We currently lack an understanding of how the digestion phase is regulated in poststroke phagocytosis, and a better understanding of how digestion is regulated and impacts outcomes following stroke has important implications for novel therapeutic strategies for stroke. As we discussed above, lysosomes theoretically play essential roles in the digestion phase. TFEB, as the key master regulator of lysosome biogenesis, may essentially contribute to digestion during poststroke phagocytosis. More research will be needed to explore how TFEB regulates poststroke phagocytosis and how TFEB impacts brain injury repair via phagocytic mechanisms following stroke. Rab7 is primarily associated with late endosome structure and possibly is the only lysosomal Rab protein identified thus far (Zhang et al., 2009). Notably, a Rab7 antagonist ameliorates brain atrophy and improves neurologic deficits following ischemic stroke (Qin et al., 2019). Therefore, future research is needed to investigate whether Rab7 plays a role in regulating digestion following stroke. Other mechanisms, such as mechanisms underlying phagolysosomal assembly (Doran et al., 2020), may also markedly modulate the digestion phase and thus have significant impacts on poststroke phagocytosis. For instance, NADPH oxidase 2-derived superoxides have been reported to be essential for conjugating the autophagy-related protein LC3-II to the phagosomal membrane. The process, in turn, promotes phagolysosomal assembly and acidification, thereby facilitating digestion of phagocytic cargos (Bagaitkar et al., 2018). Thus, future research is needed to investigate how these signaling pathways impact stroke outcomes by modulating phagocytic digestion.

### 7.2 How is continual phagocytosis regulated following stroke?

Compared to the large number of dead cells generated under pathological conditions, the number of phagocytes is rather limited (Park et al., 2011; Wang et al., 2017). For instance, it has been reported that a relatively small number of microglia/macrophages surround large volumes of hematoma at 3 days following ICH (Yan et al., 2022a). Efficient phagocytosis critically depends on the capacity of a single phagocyte to continually take up dead cells (Park et al., 2011; Wang et al., 2017; Mehrotra and Ravichandran, 2022). Thus, continual phagocytosis has significant impacts on stroke outcomes. However, how continual phagocytosis is regulated following hemorrhagic and ischemic stroke is extremely understudied. There are several challenges faced by phagocytes undergoing continual phagocytosis. First, each round of phagocytosis internalizes a substantial amount of plasma membrane as the phagosome enters the cell. Therefore, rapid restoration of the cell membrane is a prerequisite for the subsequent round of phagocytosis (Doran et al., 2020). Several mechanisms have been implicated to be essential for membrane restoration following phagocytosis. In macrophages undergoing continual phagocytosis, mitochondrial fission enhanced by elevated levels of the mitochondrial fission protein dynamin-related protein 1 leads to the restoration of the cell membrane by facilitating membrane recycling via promotion of phagolysosome-to-plasma membrane vesicular transport (Wang et al., 2017). The RAB17-dependent mechanism is also engaged in vesicular transport from phagolysosomes to recycling endosomes, which may contribute to the restoration of the plasma membrane (Yin et al., 2019). However, whether these mechanisms are also essential for continual phagocytosis following stroke or whether other novel mechanisms are engaged in poststroke phagocytosis needs further investigation. Second, degradation of engulfing material tremendously enhances metabolic cargo in phagocytes, including amino acids, lipids and nucleic acids, which must be processed in a safe and rapid manner (Han and Ravichandran, 2011; Doran et al., 2020). Mitochondria are essential for metabolism of metabolic loads. However, degradation of dead cells only results in a transient increase in the mitochondrial membrane potential (MMP) in phagocytes (Park et al., 2011), suggesting that a mechanism is activated to reduce excessive MMP. Enhanced expression of the mitochondrial uncoupling protein UCP2 was found to be the mechanism underlying MMP modulation during phagocytosis (Park et al., 2011). Notably, MMP was persistently high in phagocytes with UCP2 knockdown, and consequently, continual phagocytosis was impaired in these phagocytes. Although the signaling mechanisms linking UCP2-mediated MMP modulation to continual phagocytosis remain to be determined, the findings suggest a critical role of UCP2 in modulating continual phagocytosis. Indeed, the mitochondrial uncoupler-induced decrease in MMP has been shown to be protective following ICH (Pan et al., 2020). More relevantly, a recent study uncovered a new mechanism through which UCP2 activity rather than UCP2 expression was enhanced to promote continual phagocytosis of extraverted erythrocytes following ICH (Yan et al., 2022a). ICH was found to enhance the expression of the H_2_S synthase cystathionine β-synthase (CBS) and CBS-derived H_2_S in brain-resident phagocytic microglia (Yan et al., 2022a). Then, oxidation of CBS-derived endogenous H_2_S by sulfide-quinone oxidoreductase initiates reverse electron transfer at mitochondrial complex I, resulting in enhanced superoxide production. Complex I-derived superoxide subsequently activates uncoupling protein 2 (UCP2) to decrease MMP after erythrocytosis, which promotes continual phagocytosis and hematoma clearance following ICH. The study suggested a regulatory mechanism underlying continual phagocytosis following stroke. However, our current understanding of the mechanisms underlying continual phagocytosis is rather limited. Therefore, more studies are needed to explore the regulatory mechanisms underlying continual phagocytosis following stroke. A better understanding of the mechanisms will advance the development of novel therapeutic strategies for treating stroke (Figure 3).

### 7.3 Mechanisms involved in phagocytosis-induced phenotypic switching of phagocytes

Phagocytosis resolves inflammatory responses and activates prorepair processes under pathological conditions. The proresolving effects of phagocytosis are attributed not only to the clearance of dead cells and consequent prevention of the release of potentially cytotoxic contents from dead/dying cells (Mehrotra and Ravichandran, 2022). Emerging evidence suggests that phagocytosis itself induces the proresolving and prorepair phenotype switch of professional phagocytes, such as macrophages, under pathological conditions. When the phenotype switch induced by phagocytosis is impaired, tissue repair and functional recovery are compromised, leading to impaired resolution of inflammation and development of disease. For instance, phagocytic clearance of dying cells directly induces the transition of cardiac macrophages into a phenotype with enhanced expression of vascular endothelial growth factor (VEGF) C, and macrophage-derived VEGFC inhibits inflammation after myocardial infarction (Glinton et al., 2022) (Figure 3). Mechanistically, metabolic reprogramming is the mechanism involved in the phenotypic switch during phagocytosis. Phagocytosis is reported to enhance interleukin-10 (IL-10) expression in macrophages after myocardial infarction, which helps resolve inflammation and promote repair (Zhang et al., 2019) (Figure 3). The elevation of interleukin-10 (IL-10) is independent of glycolysis but is bolstered by fatty acids derived from apoptotic cells, which are metabolized through mitochondrial β-oxidation and the mitochondrial electron transport chain to increase the coenzyme NAD^+^. Loss of IL-10 due to the defect in mitochondrial complex III can be rescued by adding NAD^+^ precursors. NAD^+^, in turn, acted through the sirtuin signaling cascade to activate the IL-10 transcription factor PBX1. These results suggest that digestion of phagocytic cargos and subsequent metabolism of metabolic loads by phagocytes induces phenotypic switching of phagocytes via metabolic reprogramming. Phagocytosis-induced phenotypic polarization of monocyte-derived macrophages (MDMs) also contributes to functional recovery and tissue repair after ICH. By profiling the transcriptional profiles of monocyte-derived macrophages in the mouse brain following ICH, phenotypic changes in infiltrating monocyte-derived macrophages were confirmed to be essential for hematoma clearance and neurological recovery (Chang et al., 2018). Notably, phagocytosis of erythrocytes with exposed phosphatidylserine directly modulated the phenotype of both murine and human MDMs. In mice, loss of the phagocytic receptor tyrosine kinases AXL and MerTK not only reduced phagocytosis but also decreased alternative activation of macrophages after ICH, which resulted in delayed hematoma clearance and neurological recovery. Thus, phagocytosis of apoptotic erythrocytes is responsible for macrophage phenotype switching and neurological recovery following ICH. However, the molecular mechanisms underlying the phenotypic remodeling of phagocytes following stroke are not clear. Mitochondria are the key organelles for processing phagocytic loads, and current research suggests that metabolic remodeling induced by mitochondrial catabolism of metabolic loads derived from phagocytosed cargos is essential to the phenotypic polarization of phagocytes after cardiac ischemia (Zhang et al., 2019). Thus, future research is needed to explore whether the phenotypic switch of phagocytes following stroke is mediated by metabolic reprogramming and to identify key master regulators of phenotypic switching following stroke. In particular, further research is needed to explore which specific phagocyte phenotypes induced by phagocytosis confer protective and prorepair effects and how to manipulate phagocyte phenotypes to promote recovery following stroke. A better understanding of these mechanisms will advance the development of novel therapeutic strategies for treating stroke. In addition, we want to emphasize that the phagocytosis-induced phenotypic switch is related to the degradation of dead cells and subsequent mitochondrial oxidation of metabolic loads (Chang et al., 2018). Thus, the strategy of using non-degradable beads in phagocytosis assays is not suitable for the investigation of poststroke phagocytosis, especially for the investigation of phenotypic switching. We and others have used apoptotic erythrocytes in assays of microglial phagocytosis *in vitro* models of intracerebral hemorrhage (Chang et al., 2018; Yan et al., 2022a). We recommend that using ingestible dead cells is more appropriate to measure the phagocytic function of microglia than using inert latex beads.

### 7.4 The effects of aging and autophagy on phagocytosis and differential roles of peripheral and brain-resident phagocytes following stroke

There are other knowledge gaps in the understanding of poststroke phagocytosis. For instance, it has long been recognized that phagocytosis function deteriorates during aging. In particular, CD22, as a negative regulator of phagocytosis, is reported to be upregulated in aged microglia. CD22 mediates the antiphagocytic effect of α2–6-linked sialic acid, and CNS delivery of a CD22 function-blocking antibody promotes the clearance of myelin debris, amyloid-β oligomers, and α-synuclein fibrils *in vivo* by reprogramming microglia toward a homeostatic transcriptional state (Pluvinage et al., 2019). However, it remains to be investigated how aging-related deterioration of microglial phagocytosis function impacts stroke outcomes and what are the key mediators that play essential roles in aging-related deterioration of microglial phagocytosis following stroke.

In addition, autophagy, a major intracellular degradative pathway, is relatively similar to phagocytosis. Although the goal of autophagy is to degrade intracellular cargo rather than extracellular cargo, autophagy and phagocytosis share some mechanisms and cellular machinery. Interestingly, recent studies have reported that autophagy is directly relevant to phagocytosis after stroke. Basal autophagy was reported to be critical for maintaining microglial physiology, including phagocytosis. Notably, the autophagy inducer rapamycin partially prevented the phagocytosis impairment induced by ischemic stroke *in vivo* (Beccari et al., 2023). However, the mechanisms by which autophagy modulates phagocytosis following stroke warrant further investigation.

Notably, both peripheral-infiltrating myeloid cells and brain-resident microglia are involved in debris clearance via phagocytosis following stroke, and some controversial results may stem from the overlapping functions of peripheral myeloid cells and microglia. Whether microglia or macrophage phagocytosis is dominant following stroke is controversial. For instance, compared to microglia, infiltrating monocyte-derived macrophage (MDM)-mediated phagocytosis is reported to play a predominant role in pathology and recovery following stroke (Park et al., 2022). Moreover, it has been shown that MDMs have higher phagocytic activity and likely play a more important role in erythrophagocytosis following ICH (Chang et al., 2021). However, it is also reported that brain-resident microglia are the dominant phagocytes responsible for erythrophagocytosis following ICH and SAH (Schallner et al., 2015; Yan et al., 2022a). However, most studies do not distinguish between microglial and macrophage phagocytosis following stroke. Thus, more research is needed to distinguish the phagocytic roles of microglia and macrophages and to explore whether the phagocytic functions of microglia/macrophages can be individually manipulated. In addition, it also remains unclear how professional phagocytes and non-professional phagocytes cooperate to perform phagocytic functions following stroke. These issues have been reviewed (Jia et al., 2021; Chen et al., 2022; Yu et al., 2022), and we did not discuss them in detail here.

In conclusion, a number of phagocytic signals orchestrate the regulation of phagocytosis following stroke, and poststroke phagocytosis exerts multifaceted effects on stroke outcomes (Table 1), which are context-dependent and perhaps cell type dependent. Consequently, it represents a challenge to balance these effects to establish a milieu that is favorable for brain repair and functional recovery. Although much has been learned about the molecular mechanisms governing the sense and recognition of dead cells by phagocytes following stroke, some key areas remain extremely understudied, including the mechanisms involved in digestion regulation, continual phagocytosis and phagocytosis-induced phenotypic switching following stroke. Understanding these knowledge gaps holds promise for uncovering new biological targets for stroke treatment.

**TABLE 1:** Examples of molecular targets in poststroke phagocytosis and their impacts on stroke pathogenesis.

Targets	Models and interventions	Impact on pathogenesis	References
LPC (find-me signal)	Clinical samples from stroke patients and cultured microglia; blocking LPC receptors GPCR132 and P2X_7_ with antibodies	Chemoattracting microglia	Inose et al. (2015)
CX3CL1 (find-me signal)	tMCAO in mice; CX3CL1 receptor knockout	Augmenting injury and mortality	Soriano et al. (2002)
S1P (find-me signal)	tMCAO in mice; knockout or inhibition of SIP_2_ receptor	Promoting microglial M1 polarization	Sapkota et al. (2019)
S1P (eat-me signal)	tMCAO in mice and rats; supplementing S1P analog and deleting the novel S1P receptor TREM2 in mice	Promoting microglial phagocytosis of dead neurons and reducing infarcts	Xue et al. (2022)
Complement C3a (find-me signal)	Permanent MCAO in mice and BCCAO in rats; antagonist or knockout of C3a receptor	Inducing excessive phagocytosis and ischemic injury	Surugiu et al. (2019), Zhang et al. (2020a)
ATP (find-me signal)	BCCAO in mice; knockout of ATP receptor P2Y_12_	Promoting neuroinflammation and inducing neurotoxicity	Webster et al. (2013)
PS (eat-me signal)	tMCAO in rats; knockdown of TMEM16F (the scramblase responsible for PS exposure)	Promoting microglial phagocytosis and aggravating neuronal loss and deficits	Zhang et al. (2020a)
PS (eat-me signal)	ICH in mice and cultured macrophages or microglia; blocking PS with annexin V	Promoting erythrocyte phagocytosis of by macrophages and microglia	Chang et al. (2018), Yan et al. (2022a)
MFG-E8 (opsonin)	Bilateral injection of endothelin-1 in rodents; MFG-E8 knockout	Aggravating delayed neuronal loss and motor deficits	Neher et al. (2013)
MerTK (phagocytic receptor)	Bilateral injection of endothelin-1 in rodents; MerTK knockout	Aggravating delayed neuronal loss and motor deficits	Neher et al. (2013)
C1q (opsonin)	Unilateral hypoxia-ischemia in neonatal rats; reducing C1q with a classic components pathway inhibitor	Aggravating brain damage	Kumar et al. (2021)
Complements (opsonin)	tMCAO in mice; targeted complement inhibition in the ischemic area	Promoting phagocytosis of stressed but viable neurons	Alawieh et al. (2018)
C3a (opsonin)	Unilateral hypoxia-ischemia in neonatal mice; C3a overexpression, C3a peptide administration and knockout of C3a receptor (CR3)	Ameliorating tissue loss and memory impairment	Jarlestedt et al. (2013)
CD36 (phagocytic receptor)	ICH in rodents; CD36 knockout and pharmacologically enhancing CD36 expression	Promoting erythrocyte phagocytosis and hematoma resolution	Fang et al. (2014), Chang et al. (2020)
CD36 (phagocytic receptor)	tMCAO in mice; CD36 knockout and pharmacological inhibition	Aggravating a cuteinjury and neurological deficits; mediating phagocytosis in the recovery phase	Cho et al. (2005), Woo et al. (2016)
CD47 (do not eat-me signal)	tMCAO in mice; knockout of CD47 or its receptor SIRPα	Aggravating acute ischemic injury	Jin et al. (2009), Wang et al. (2012)
CD47 (do not eat-me signal)	ICH in mice or IVH in rats; deletion of CD47 in erythrocytes and blocking CD47 with an antibody	Inhibiting erythrocyte phagocytosis and delaying hematoma resolution	Jin et al. (2009), Jing et al. (2019), Tao et al. (2020), Ye et al. (2021)
Siglec-E (anti-phagocytic receptor)	tMCAO in mice and OGD in primary neurons; knockout of Siglec-E	Reducing neuronal death and ischemic injury	Li et al. (2022)
CD300a (anti-phagocytic receptor)	tMCAO in mice; knockout of CD300a or blocking CD300a with an antibody	Aggravating brain damage and neurological deficits	Nakahashi-Oda et al. (2021)
TFEB (activator of digestion)	tMCAO in rats; activating TFEB with a specific activator or by GSK-3β inhibition	Reducing ischemic injury	Wu et al. (2021), Zhang et al. (2022)
PPARγ and Nrf2 (Regulator of phagocytic receptor expression)	ICH in rodents; PPARγ and Nrf2 activators	Promoting CD36 expression, erythrocyte phagocytosis and hematoma resolution	Zhao et al. (2007), Ballesteros et al. (2014), Zhao et al. (2015), Li et al. (2021)
Endogenous CO (Regulator of phagocytic receptor expression)	SAH in mice; knockout of the CO synthase HO-1 and supplementing exogenous CO	Promoting CD36 expression, erythrocyte phagocytosis, hematoma resolution and functional recovery	Schallner et al. (2015), Kaiser et al. (2020)
Endogenous H_2_S (Regulator of continual phagocytosis)	ICH in mice; knockout of the H_2_S synthase CBS and supplementing exogenous H_2_S	Promoting continual phagocytosis of erythrocytes, hematoma resolution and functional recovery	Yan et al. (2022a)

## Figures and Tables

**Table:** 

**5-LO**	5-lipoxygenase
**ABCA1**	ATP-binding cassette transporter A1
**AMPK**	AMP-activated protein kinase
**BCCAO**	Bilateral common carotid artery occlusion
**CBS**	Cystathionine β-synthase
**CO**	Carbon monoxide
**CR3**	The complement C3a receptor
**Gas6**	Growth arrest-specific gene 6
**GPCR**	G-protein coupled receptor
**H2S**	Hydrogen sulfide
**ICH**	Intracerebral hemorrhage
**IL-4 (10)**	Interleukin-4 (10)
**IVH**	Intraventricular hemorrhage
**LCN2**	Lipocalin-2
**LPC**	Lysophosphatidylcholine
**LRP1B**	Low-density lipoprotein receptor related protein 1B
**MerTK**	Tyrosine protein kinase Mer
**MFG-E8**	Milk fat globule-epidermal growth factor 8
**MMP**	Mitochondrial membrane potential
**Nrf2**	Nuclear factor-erythroid 2 p45-related factor 2
**P2X7**	P2X purinoreceptor 7
**P2X12**	P2X purinoreceptor 12
**PPARγ**	Peroxisome proliferator-activated receptor-γ
**LRP1**	Low-density lipoprotein receptor-related protein 1
**PS**	Phosphatidylserine
**RBC**	Red blood cells
**S1P**	Sphingosine-1-phosphate
**S1P2**	S1P receptor 2
**SAH**	Subarachnoid hemorrhage
**Siglec**	Sialic acid-binding immunoglobulin-like lectins
**SIRPα**	Signal-regulatory protein-α
**SQR**	Sulfide-quinone oxidoreductase
**ST2**	IL-1 receptor-like 1
**STAT6**	Signal transducer and activator transcription 6
**TFEB**	Transcription factor EB
**UCP2**	Uncoupling protein 2
**VEGF**	Vascular endothelial growth factor.
